# Prevalence and clinical impact of Vitamin A Deficiency (VAD) in critically ill children with sepsis

**DOI:** 10.1186/s12887-025-06143-0

**Published:** 2025-10-13

**Authors:** Amany Mohammed El-Rebigi, Ahmed Shaheen Dabour, Sara Hosny El-Farargy, Amr Ahmed Aly Ibrahim, Ola Samir El-Shimi, Rasha Mohammed Zakaria

**Affiliations:** 1 Pediatric and Neonatology Department, Faculty of Medicine, Benha University, Benha, Egypt; 2 Faculty of Medicine, Benha University, Benha, Egypt; 3 Faculty of Medicine, Monoufyia University, Monoufyia, Egypt; 4 Clinical and Chemical Pathology Department, Faculty of Medicine, Benha University, Benha, Egypt

**Keywords:** Vitamin A deficiency, Sepsis, Critically ill children, Pediatric intensive care unit

## Abstract

**Background:**

Data on vitamin A deficiency (VAD) among critically ill pediatric patients with sepsis and its impact remain limited. This study aimed to determine the prevalence of VAD in septic children and evaluate its association with clinical outcomes.

**Methods:**

This prospective cohort included 180 children with sepsis admitted to the Pediatric Intensive Care Unit (PICU) of Benha University Hospital, and 40 healthy controls. Using ELISA, serum vitamin A (VA) levels were measured upon admission. Clinical parameters, including the Pediatric Risk of Mortality (PRISM) scores, sepsis progression, ventilation duration, PICU stay, and mortality were assessed. Multivariate regression evaluated the relationship between VAD and sepsis severity.

**Results:**

VAD was significantly more prevalent among septic patients than controls (61.7% vs. 20%, *P* < 0.001). ROC analysis identified serum VA ≤ 21.4 µg/dl as a significant predictor of sepsis (AUC = 0.699, *P* < 0.001). While VAD was more frequent in non-survivors (71.4%), the association with mortality was not statistically significant. VAD was linked to higher PRISM scores, longer PICU stays, and greater sepsis severity.

**Conclusion:**

Our cohort found that VAD is more prevalent in pediatric sepsis patients compared to controls and correlated with higher PRISM scores, sepsis severity and prolonged stays in PICUs. Nevertheless, VAD did not demonstrate a statistically significant association with 28-days mortality. These findings highlight the necessity for additional large-scale research to determine if routine screening of VAD and potential supplementation could improve outcomes in these patients.

**Supplementary Information:**

The online version contains supplementary material available at 10.1186/s12887-025-06143-0.

## Introduction

Vitamin A (VA) and its lipid-soluble metabolites are predominantly detected in serum bound to specific cellular-binding proteins [1]. Its biological functions are diverse and essential, encompassing the maintenance of visual function, promotion of cellular growth and reproduction, embryogenesis, preservation of epithelial and mucosal integrity, and modulation of immune responses [2]. The pleiotropic effects of VA on multiple physiological systems emphasize the critical need for adequate VA status to ensure cellular homeostasis and host defence.

Vitamin A deficiency (VAD) continues to be a pressing global nutritional concern, arising primarily from inadequate dietary intake, impaired gastrointestinal absorption, or elevated metabolic demands [3]. Globally, the prevalence of VAD among children under five years is estimated to range between 30% and 35% [4], with the highest burden reported in Southeast Asia and sub-Saharan Africa. VAD contributes significantly to preventable morbidity and mortality, accounting for approximately 500,000 cases of pediatric blindness annually and markedly increasing the risk of severe infections [5]. Recognizing these risks, population-based vitamin A supplementation (VAS) programs have been advocated, particularly in low-resource settings, demonstrating a modest but consistent reduction in under-five mortality by 5–15% [4].

Beyond its traditional roles, VA has significant effects on the immune system. VAD impairs both innate and adaptive immunity through mechanisms such as defective phagocytic activity, weakened epithelial barrier integrity, and T-cell dysregulation [6, 7]. Retinoic acid (RA), the active metabolite of VA, plays a crucial role in the differentiation and functional regulation of immune cells. RA enhances macrophage and neutrophil maturation, supports the cytotoxic activity of natural killer T cells, and helps regulate a balance between pro-inflammatory and anti-inflammatory T cell subsets [8–10]. Specifically, RA promotes the development of regulatory T cells while suppressing interleukin-6-driven pro-inflammatory Th17 cells, which may help maintain immune homeostasis during systemic inflammation [10].

Given these immunomodulatory properties, it is plausible that VAD may contribute to adverse outcomes in conditions characterized by severe immune dysregulation—such as sepsis. Despite the high global prevalence of VAD, its potential role in the pathogenesis or clinical course of sepsis, particularly in pediatric critical care settings, remains underexplored.

Sepsis, defined as a dysregulated host response to infection resulting in life-threatening organ dysfunction, remains a leading cause of pediatric intensive care unit (PICU) admission and global mortality [11]. An estimated 19% of all deaths worldwide are attributed to sepsis, with the greatest burden falling upon children under the age of five [12, 13]. The disease course of sepsis is typically biphasic—initially characterized by an exaggerated proinflammatory response, followed by an immunosuppressive phase that renders patients vulnerable to secondary infections and opportunistic pathogens [14, 15]. These immune alterations are strikingly like those observed in VAD, suggesting a potential interaction between nutritional status and sepsis progression.

Although the immunological and epidemiological implications of VAD are well documented, its contribution to the severity and prognosis of pediatric sepsis has not been systematically investigated. In this context, it is imperative to evaluate whether suboptimal VA status may serve as a modifiable risk factor for adverse clinical outcomes in septic children.

Therefore, we hypothesized that suboptimal VA status is associated with increased sepsis severity and adverse clinical outcomes, a relationship that will be evaluated in our cohort study, exploring the prevalence of VAD among critically ill children with sepsis.

## Methods

### Study design and participants

This prospective cohort study was conducted at the Pediatric Intensive Care Unit (PICU) of Benha University Hospital over a one-year period, from May 2023 to April 2024. Pediatric patients aged between 29 days and 16 years who were admitted for the first time with a diagnosis of sepsis, as defined by the international pediatric sepsis consensus criteria [16], were eligible for inclusion. For comparative purposes, forty age- and sex-matched healthy children were recruited as a control group.

To minimize confounding factors and isolate the association between VAD and clinical outcomes in pediatric sepsis, the following patients were excluded: (1) children older than 16 years; (2) those with a history of recurrent hospital admissions; (3) premature infants or those with low birth weight; (4) patients with underlying primary or acquired immunodeficiency, malignancies or recent exposure to chemotherapy or corticosteroids, due to their impact on immune function, inflammation, and micronutrient metabolism; (5) patients with chronic organ dysfunction, including heart failure and hepatic or renal impairment, which can alter vitamin A metabolism and clearance; (6) those discharged without medical approval; and (7) those with missing data or loss of follow-up.

All enrolled pediatric patients received standardized nutritional support in accordance with our institutional pediatric sepsis protocol, which follows age-appropriate caloric intake, targeting 100–120 kcal/kg/day for well-nourished children and up to 150–220 kcal/kg/day in malnourished cases, along with protein targets of 1.5 gm/kg/day and micronutrient supplementation. All nutritional interventions were tailored based on daily clinical assessments and laboratory monitoring of metabolic parameters, fluid balance, and signs of feeding intolerance. Enteral nutrition was provided if the patient was hemodynamically stable and exhibited no contraindications; otherwise, parenteral nutrition was administered if the enteral nutrition was not feasible or sufficient.

The study was approved by the Research Ethics Committee of the Faculty of Medicine, Benha University (Approval Code: RC# 17–4-2023), by the Declaration of Helsinki. Informed consent was obtained from the parents or legal guardians of all participants after explaining the study objectives and procedures. The study was registered at ClinicalTrials.gov (NCT# 06391437). The reporting adhered to the Strengthening the Reporting of Observational Studies in Epidemiology (STROBE) guidelines [17].

### Data collection

At the time of PICU admission, comprehensive demographic data including age, sex, anthropometric measurements, and place of residence were recorded. Clinical assessments and laboratory investigations were conducted, encompassing vital signs, Pediatric Risk of Mortality (PRISM) scores, and relevant haematological and biochemical parameters. Inflammatory and metabolic indicators such as C-reactive protein (CRP), lactate, and plateletcrit levels (PCT) were also evaluated. The source of infection, microbiological findings, duration of mechanical ventilation (MV), and length of hospital and PICU stays were documented. Clinical progression to severe sepsis or septic shock, as well as 28-day all-cause mortality, was recorded.

### Sample size

The required sample size was calculated using Epi Info version 7.2.5.0 software, based on a previously published study by Zhang et al. (2019), which reported a 58.8% prevalence of VAD among children with sepsis [18]. With a confidence level of 95% and a margin of error of 8%, the minimum required sample size was estimated to be 145 patients. To compensate for potential missing data, incomplete records, or unforeseen exclusions, an additional 10% was added, raising the minimum sample size to approximately 160 patients. Based on this estimation, a total of 180 pediatric patients with sepsis were ultimately enrolled to ensure adequate statistical power and robustness of the findings.

### Blood sampling and vitamin a assessment

Blood samples were collected by venipuncture upon admission prior to or within the first hour of initiating intravenous fluid resuscitation for all cases, ensuring that VA levels reflect baseline nutritional status rather than dilutional effect, and before the initiation of any nutritional support. All procedures were performed using aseptic techniques. Serum VA concentrations were measured using a commercial enzyme-linked immunosorbent assay (ELISA) kit (DLR-VA-Ge). According to WHO criteria, serum VA levels below 20 μg/dl were considered deficient [5].

### Statistical analysis

Data were analysed using IBM SPSS Statistics version 28.0 (IBM Corp., Armonk, NY, USA). The Kolmogorov–Smirnov test was employed to evaluate the normality of continuous variables. Normally distributed variables were expressed as means and standard deviations, while non-normally distributed variables were summarized as medians with interquartile ranges. Categorical variables were reported as frequencies and percentages.

Comparative analyses between groups were performed using appropriate parametric or non-parametric statistical tests. The Chi-square test or Fisher’s exact test was used for categorical variables, and the independent samples t-test or Mann–Whitney U test was applied for continuous variables, depending on data distribution. Given the exploratory nature of the analyses, no formal correction for multiple comparisons was applied. The primary outcomes were predefined, and results from secondary or post-hoc comparisons should be interpreted cautiously. Receiver operating characteristic (ROC) curve analysis was conducted to assess the predictive utility of vitamin A levels for identifying sepsis, with calculation of the area under the curve (AUC), sensitivity, specificity, and predictive values. Multivariable logistic regression was used to evaluate the association between vitamin A deficiency and sepsis severity, adjusting for potential confounding variables. Adjusted odds ratios with corresponding 95% confidence intervals were reported. Statistical significance was defined as a p-value less than 0.05.

## Results

### Demographic characteristics

**Fig. 1:**
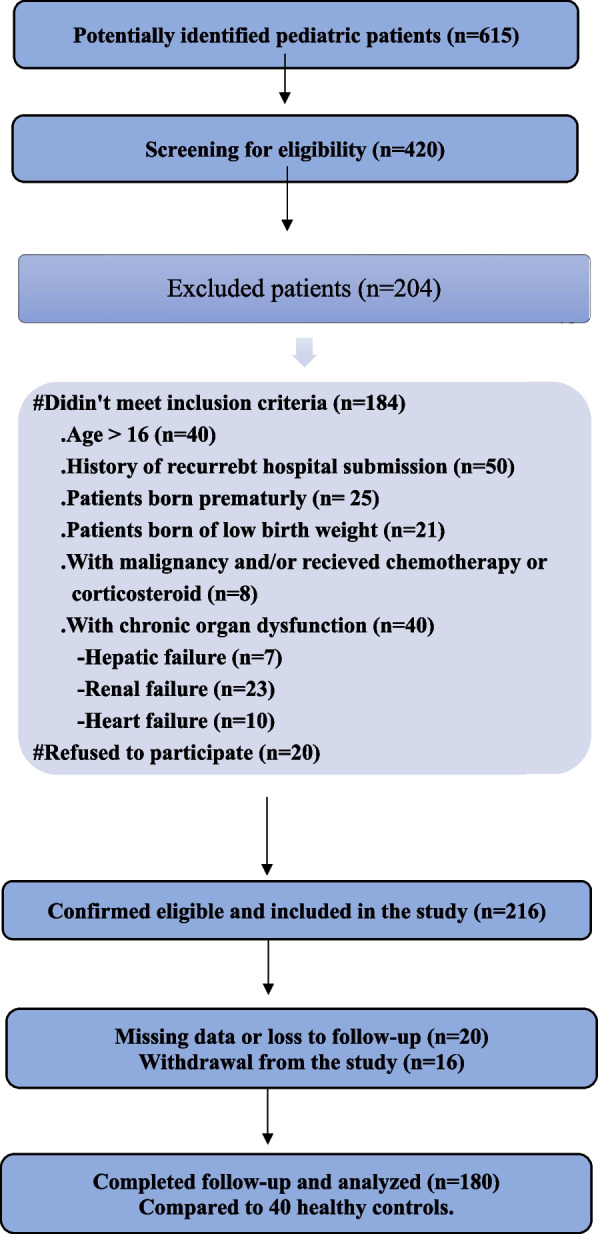
STORBE compliant flow diagram

A total of 220 children were enrolled in this study, comprising 180 pediatric patients diagnosed with sepsis and 40 healthy children who served as controls. The flow Chart is presented in Fig. 1. The demographic analysis demonstrated that the two groups were comparable in terms of age, sex distribution, weight, body mass index (BMI), and residency. No statistically significant differences were found between patients and controls regarding these baseline characteristics (P-values: age = 0.662, gender = 0.163, weight = 0.280, BMI = 0.682, and residency = 0.174), confirming adequate matching. These data are presented in Table 1.

**Table 1:** Demographic characteristics of the studied groups

	**Patients** **(*n* = 180)**	**Controls** **(*n* = 40)**	***P*-value**
Age (months)	49 (4—168)	49 (6—144)	0.662
Gender			
Male	120 (66.7)	22 (55)	0.163
Female	60 (33.3)	18 (45)	
Weight (kg)	16.9 (5.9—48)	21.8 (7—38)	0.28
BMI (kg/m^2^)	21 ± 2.2	21.2 ± 2.4	0.682
Residency			
Rural	111 (61.7)	20 (50)	0.174
Urban	69 (38.3)	20 (50)	

Data expressed as mean ± SD, median (IQR) or number (percent)

*BMI* Body mass index

### Clinical and laboratory characteristics of septic patients

Detailed clinical and laboratory assessments were conducted for all septic patients. The findings, including PRISM scores, inflammatory markers, hematological parameters, metabolic indicators, and liver and renal function tests, are summarized in Table 2. These measurements provided the basis for evaluating the severity and progression of sepsis about vitamin A status and clinical outcomes.

**Table 2:** Clinical and laboratory characteristics of the sepsis group:

Clinical characteristics	
PRISM score	12 ± 3
Temperature (°C)	38.9 ± 0.4
Hypoglycemia (mg/dl)	30 (16.7)
Lactate (mmol/L)	2.8 ± 1.1
WBCs (× 10^3^/µl)	11.17 ± 2.5
Hb (g/dl)	11.2 ± 1.2
PLTs (× 10^3^/µl)	163 ± 76
Plateletcrit (PCT) (%)	0.218 ± 0.02
CRP (mg/L)	22 (6—124)
Creatinine (mg/dl)	0.9 ± 0.4
Urea (mg/dl	17 ± 8
ALT (U/L)	54 (35—264)
AST (U/L)	60 (37—256)
Albumin (g/dl)	3.2 ± 0.6

Continuous variables are presented as mean ± standard deviation or median (range), as appropriate. Categorical variables are presented as number (percentage). Hypoglycemia is the only categorical variable in this table and is shown as n (%)

Data expressed as mean ± SD, median (IQR) or number (percent)

*PRISM* Pediatric Risk of Mortality, *WBCs* White blood cells, *Hb* Hemoglobin, *PLTs* Platelets, *PCT* plateletcrit, *CRP* C-reactive protein, *ALT* Alanine aminotransferase, *AST* Aspartate aminotransferase

### Serum vitamin a levels and deficiency status

**Fig. 2:**
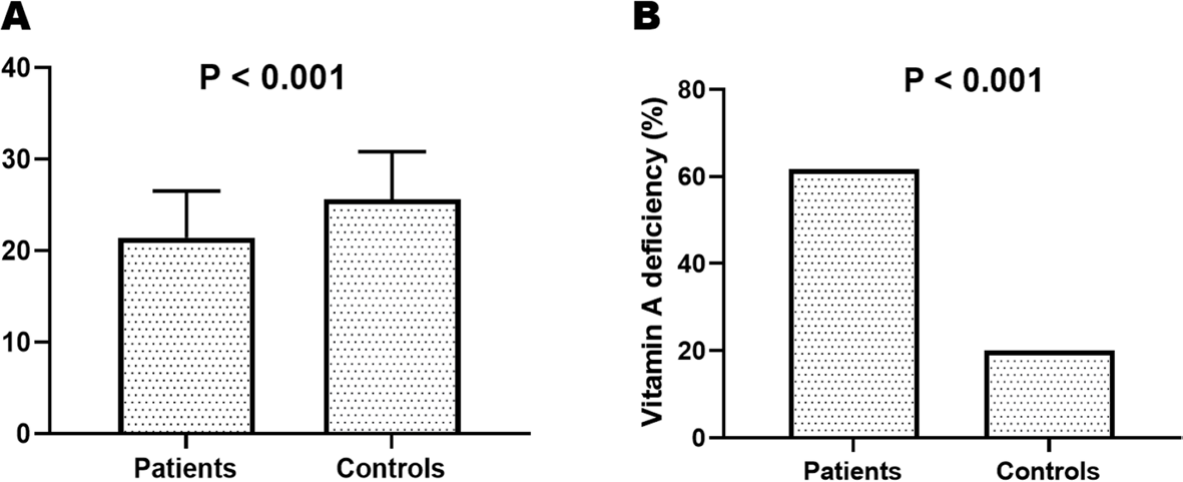
**A** Serum VA levels among the studied groups **B** Prevalence of VAD in studied groups

Vitamin A levels were significantly lower in septic patients compared to healthy controls. The mean serum VA concentration in the sepsis group was 21.4 ± 5.1 µg/dl, whereas the control group had a higher mean value of 25.6 ± 5.2 µg/dl (*P* < 0.001). In addition to this quantitative reduction, the prevalence of vitamin A deficiency, defined as serum VA < 20 µg/dl, was markedly elevated among patients with sepsis (61.7%) compared to controls (20%), a difference that was also statistically significant (*P* < 0.001). These findings are illustrated in Fig. 2A and B.

### Sepsis characteristics and clinical outcomes

Among the septic patients, the most frequently identified sources of infection were respiratory tract infections (31.7%), followed by bloodstream infections (28.3%) and central nervous system infections (10%). Blood cultures were positive in 15% of the cases (n = 27), with *Streptococcus pneumoniae* (33.3%), *Klebsiella pneumoniae* (22.2%), and *Pseudomonas aeruginosa* (22.2%) being the most isolated organisms. Regarding disease severity, 56.7% of cases were classified as early sepsis, 23.3% as severe sepsis, and 20% as septic shock. The mean duration of mechanical ventilation, average length of hospital stay, and mean PICU stay were recorded as clinical outcome parameters. The overall 28-day mortality rate among the septic cohort was 11.7%. Further details are provided in Table 3.

**Table 3:** Sepsis characteristics and outcome in the sepsis group

Sepsis characteristics &outcome	n (%)
Source of infection	
Respiratory	57 (31.7)
Blood	51 (28.3)
CNS	18 (10)
Mixed	15 (8.3)
GIT	12 (6.7)
Urine	12 (6.7)
Soft tissue	9 (5)
Others	6 (3.3)
Blood Culture	
Positive	27 (15)
Negative	153 (85)
Isolated organism	
Streptococcus pneumonia	9 (33.3)
Klebsiella pneumonia	6 (22.2)
Pseudomonas aeruginosa	6 (22.2)
Staphylococcus	3 (11.1)
Sepsis outcome	
Duration of MV (hours)	36 (5—156)
Length of hospital stay (days)	12 ± 4
Length of PICU stay (days)	7 ± 3
Degree of sepsis	
Early	102 (56.7)
Severe	42 (23.3)
Shock	36 (20)
28- day mortality	21 (11.7)

Data expressed as mean ± SD, median (IQR) or number (percent)

*CNS* Central nervous system, *GIT* Gastrointestinal tract, *MV* Mechanical ventilation, *PICU* Pediatric intensive care unit

### Predictive value of vitamin a for sepsis

**Fig. 3:**
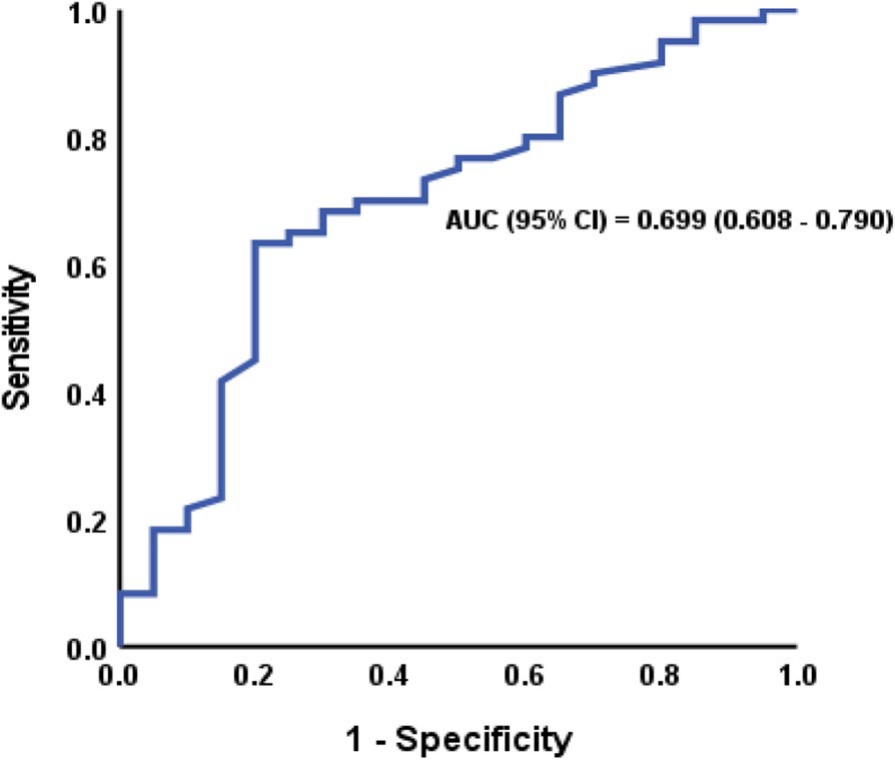
ROC analysis of vitamin A in predicting sepsis

Receiver operating characteristic (ROC) curve analysis was performed to assess the diagnostic value of vitamin A levels in predicting sepsis. Serum VA concentrations < 21.4 µg/dl demonstrated fair performance in predicting pediatric sepsis. The area under the curve (AUC) was 0.699 (95% CI: 0.608–0.790, *P* < 0.001), with a sensitivity of 63.3%, specificity of 80%, positive predictive value (PPV) of 93.4%, and negative predictive value (NPV) of 32.7%. This is depicted in Fig. 3.

### Correlation of vitamin a with clinical and laboratory parameters

**Fig. 4:**
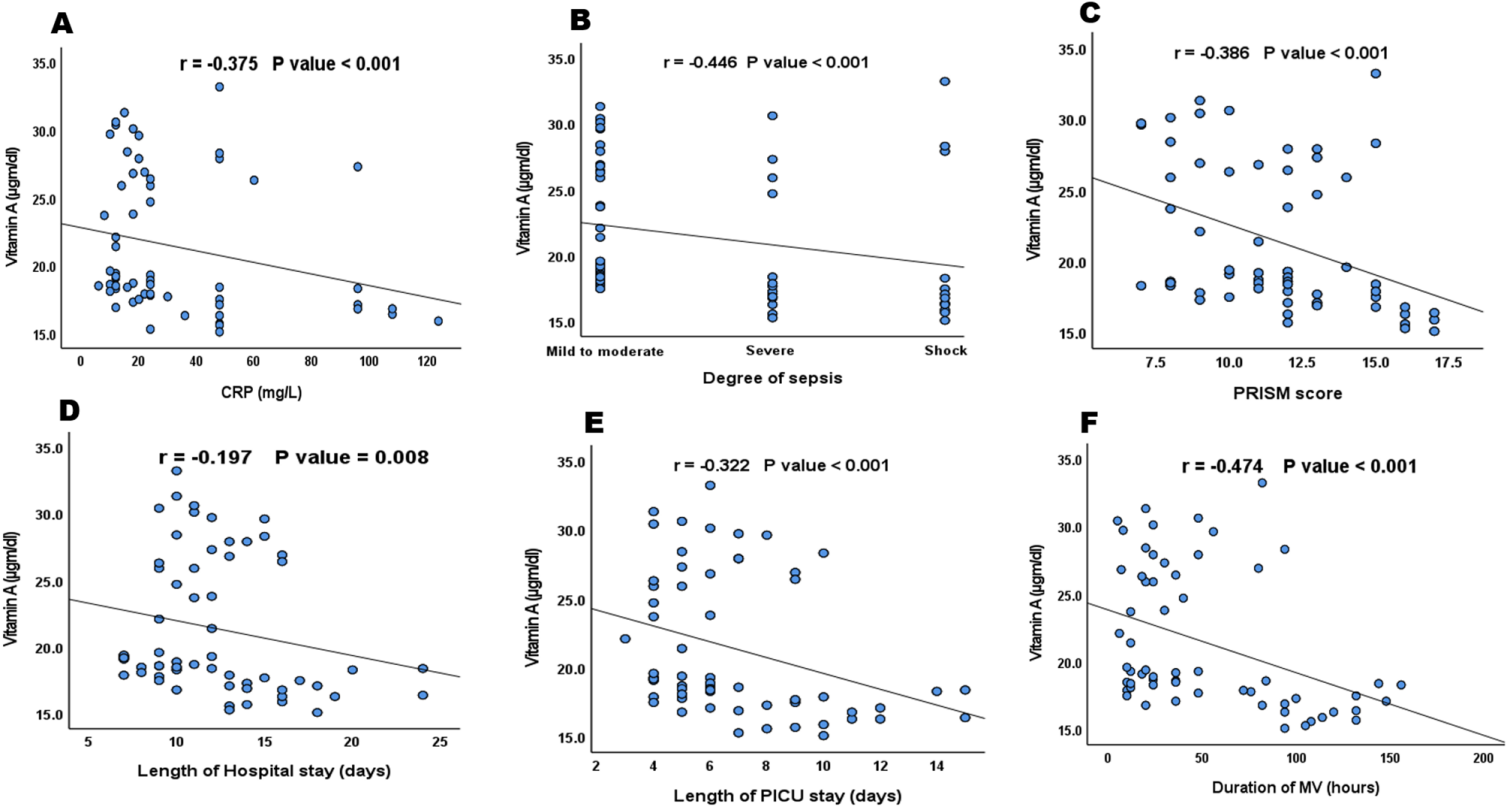
Correlation between VA level and **A** CRP; **B** Degree of sepsis; **C** PRISM; **D** Length of hospital stay; **E** Length of PICU stay and **F** Duration of MV

Correlation analysis revealed significant inverse relationships between serum vitamin A levels and several key clinical parameters. These included C-reactive protein (CRP), sepsis severity, PRISM score, hospital stay duration, PICU stay, and the duration of mechanical ventilation. In contrast, a positive correlation was observed between vitamin A levels and serum albumin (*r* = 0.288, *P* < 0.001). No statistically significant correlations were found between VA levels and patients’ age (*r* = 0.121, *P* = 0.105), weight (*r* = 0.051, *P* = 0.498), BMI (*r* = 0.043, *P* = 0.569), alanine aminotransferase (ALT) (*r* = 0.006, *P* = 0.94), or aspartate aminotransferase (AST) (*r* = –0.053, *P* = 0.479). These relationships are shown in Fig. 4A–F.

### Clinical and laboratory features according to vitamin a status

To further evaluate the impact of vitamin A on disease manifestation, patients were stratified into two groups based on serum vitamin A levels: vitamin A-deficient (VAD) and sufficient vitamin A (SVA). Comparison of clinical characteristics and laboratory values between the two groups demonstrated significant associations between VAD and adverse clinical parameters. These included higher sepsis severity, lower platelet counts, elevated PRISM scores, higher body temperatures, elevated lactate and WBC levels, prolonged ventilator dependency, and extended PICU stays. These results are summarized in Table 4.

**Table 4:** Clinical and laboratory characteristics according to vitamin A deficiency

Variable	VAD (*n* = 111)	SVA (*n* = 69)	*P*-value
Hypoglycemia, n (%)	24 (21.6)	6 (8.7)	0.024
Positive blood culture, n (%)	18 (16.2)	9 (13.0)	0.562
Degree of sepsis – Early, n (%)	54 (48.6)	48 (69.6)	0.021
Degree of sepsis – Severe, n (%)	30 (27.0)	12 (17.4)	
Degree of sepsis – Shock, n (%)	27 (24.3)	9 (13.0)	
CRP (mg/L), median (IQR)	24 (6–124)	18 (8–96)	0.055
PLTs (× 10^3^/µl), mean ± SD	136 ± 58	206 ± 83	< 0.001
PRISM score, mean ± SD	12 ± 3	11 ± 2	< 0.001
Temperature (°C), mean ± SD	39.0 ± 0.4	38.7 ± 0.4	< 0.001
Albumin (g/dl), mean ± SD	3.1 ± 0.6	3.5 ± 0.6	< 0.001
WBCs (× 10^3^/µl), mean ± SD	11.68 ± 2.78	10.36 ± 1.67	< 0.001
Lactate (mmol/L), mean ± SD	3.1 ± 1.2	2.2 ± 0.6	< 0.001
Duration of MV (h), median (IQR)	48 (10–156)	24 (5–94)	< 0.001
Need for vasoactive–inotropic support, n (%)	**49 (44.1)**	**15 (21.7)**	**0.003**
Vasoactive–Inotropic Score (VIS), median (IQR)	**12 (6–18)**	**8 (4–12)**	**0.004**
Length of PICU stay (days), mean ± SD	8 ± 3	6 ± 2	< 0.001
Length of hospital stay (days), mean ± SD	13 ± 5	12 ± 2	0.077
28-day mortality, n (%)	15 (13.5)	6 (8.7)	0.328

Data represented as mean ± SD, median (IQR) or number (percent)

*VAD* Vitamin A Deficiency, *SVA* sufficient Vitamin A, *CRP* C-reactive protein, *PLTs* Platelets, *PRISM* Pediatric Risk of Mortality, *WBCs* White Blood Cells, *MV* Mechanical Ventilation, *PICU* Pediatric Intensive Care Unit

### Vitamin a levels and mortality outcomes

**Fig. 5:**
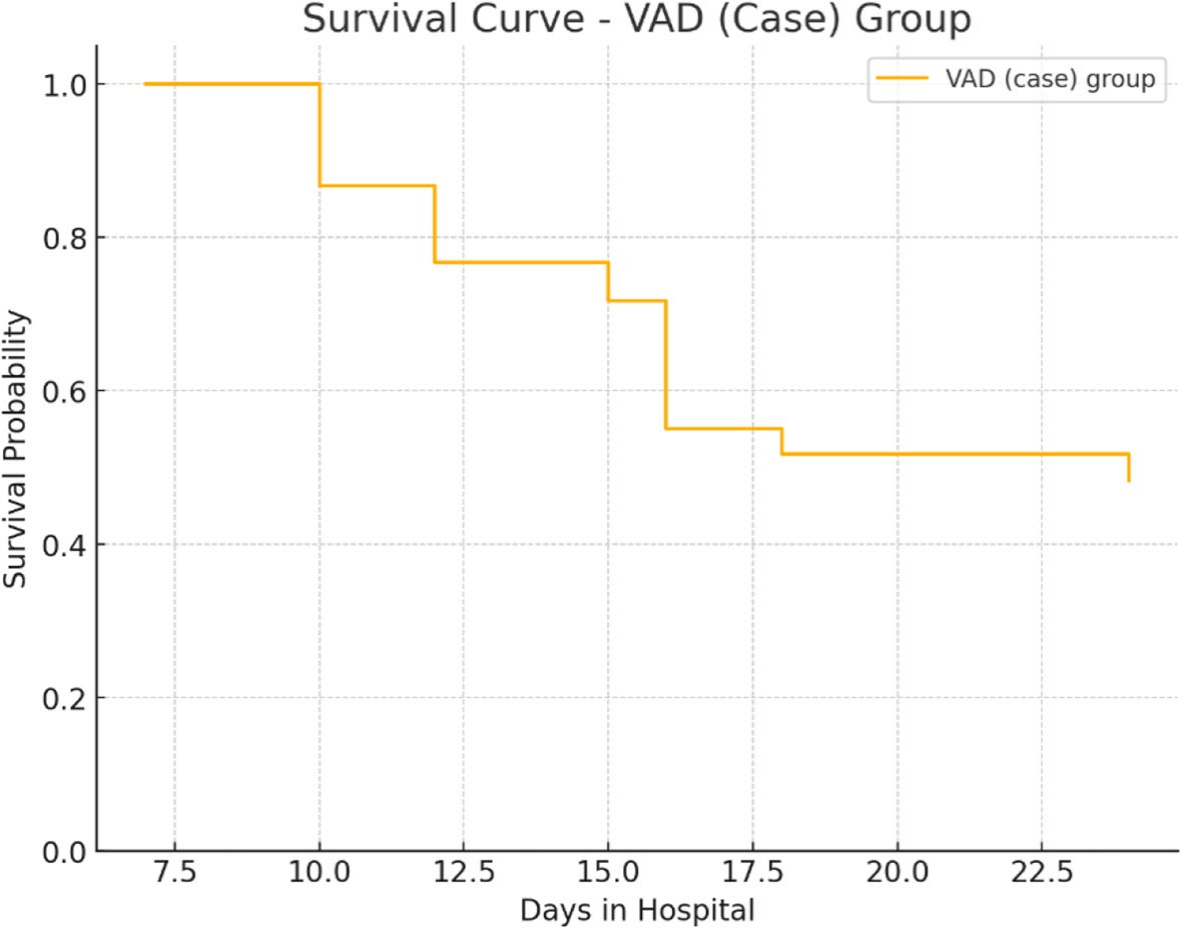
Survival curve (VAD) group

Although non-survivors had lower mean vitamin A levels (19.8 ± 5.2 µg/dl) compared to survivors (21.6 ± 5.1 µg/dl), the difference did not reach statistical significance (*P* = 0.134). Similarly, the prevalence of VAD was higher among non-survivors (71.4%) than survivors (60.4%), but this difference was not statistically significant (*P* = 0.328). These findings suggest a trend toward worse outcomes in VAD patients, although the study was not powered to detect a mortality difference. Survival analysis was presented in Fig. 5.

### Predictors of sepsis

Multivariate logistic regression analysis was conducted to evaluate the association between vitamin A levels and the risk of sepsis, after adjusting for potential confounding variables including age, sex, BMI, and residency. The analysis demonstrated that each one-unit increase in serum vitamin A level was associated with a 13.6% reduction in the risk of sepsis (OR = 0.864, 95% CI: 0.806–0.927, *P* < 0.001), indicating a strong inverse relationship. Results are provided in Table 5.

**Table 5:** Multivariable logistic regression model to predict sepsis

	**OR (95% CI)**	***P*-value**
Vitamin A (µgm/dl)	0.864 (0.806—0.927)	** < 0.001**
Age (month)	1.007 (0.998—1.017)	0.132
Sex	0.843 (0.389—1.826)	0.665
BMI (kg/m^2^)	0.993 (0.831—1.187)	0.940
Residency	0.709 (0.326—1.539)	0.384

*OR* Odds ratio, *CI* Confidence interval, *BMI* Body mass index

## Discussion

This study provides novel evidence on the high prevalence of vitamin A deficiency (VAD) among critically ill pediatric patients with sepsis and its association with adverse clinical outcomes. Our findings support a growing body of evidence implicating micronutrient status as a determinant of disease progression and prognosis in pediatric intensive care settings.

VAD was detected in 61.7% of septic children compared to 20% of healthy controls, representing a significant burden. This proportion is higher than the 11.7% reported in a Chinese cohort of children under six years [19], and aligns with global estimates indicating that up to one-third of preschool-aged children are vitamin A-deficient, particularly in Africa and Southeast Asia [5]. Such widespread prevalence, including both overt and subclinical forms, has been strongly associated with immunosuppression, recurrent infections, and preventable childhood mortality [5, 20].

The immunological importance of vitamin A is well established. VA deficiency disrupts mucosal barriers, impairs phagocyte function, and alters T-cell differentiation, thereby increasing vulnerability to infections [6, 7, 21]. In our cohort, septic children with VAD had higher illness severity scores, more frequent hypoglycemia, and a greater need for respiratory support and prolonged hospitalization. These findings mirror those of Zhang et al. [18], who similarly observed higher PRISM scores and poorer outcomes in vitamin A-deficient pediatric sepsis cases.

Notably, although non-survivors had lower mean VA levels and a higher frequency of VAD than survivors, the difference was not statistically significant. This lack of significance may reflect the limited sample size, as previous epidemiological studies have established a clear link between VAD and childhood mortality [22–24]. Additionally, the overall 28-day mortality rate in our cohort (11.7%) was higher than that reported by Wang et al. [25], likely reflecting variations in healthcare infrastructure between low- and high-resource settings.

The pathophysiological basis of these associations may be explained by the immunomodulatory effects of retinoic acid, a bioactive metabolite of VA. Retinoic acid suppresses IL-6-driven Th17 pro-inflammatory responses while enhancing regulatory T-cell development [26, 27]. This dual regulation of inflammatory pathways is critical in the context of sepsis, which often follows a biphasic trajectory of hyperinflammation followed by immune suppression [14, 15, 28]. In experimental models, RA has demonstrated anti-inflammatory effects and protection against immune-mediated tissue injury [21].

Our data also demonstrated that serum VA levels correlated negatively with markers of inflammation (CRP), disease severity (PRISM score), and organ dysfunction (lactate, ventilation time, length of stay). A positive correlation was found with albumin, suggesting a potential relationship between nutritional and inflammatory status. These findings are consistent with those of Corcoran et al. [29], who reported inverse correlations between VA and inflammatory biomarkers such as procalcitonin.

Interestingly, thrombocytopenia was more pronounced in VAD patients, highlighting a possible role of vitamin A in platelet regulation. Retinoic acid receptor α is expressed on human platelets and influences thrombopoiesis, potentially linking VAD to coagulopathy observed in severe infections [30]. Given that thrombocytopenia is an established prognostic marker in sepsis [31–33], this association warrants further investigation.

ROC analysis confirmed the potential diagnostic utility of VA, with a serum level ≤ 21.4 µg/dl demonstrating fair predictive performance for sepsis (AUC = 0.699). Although the WHO defines VAD utilizing a threshold lower than 20 µg/dl [5], our ROC analysis identified a slightly higher cut-off value (<21.4 µg/dl) as a better predictor for adverse outcomes in pediatric sepsis. This discrepancy may be attributed to the pathophysiology context of acute critical illness, where subclinical reductions of vitamin A could impact immune function and clinical trajectory, along with the relatively small sample size. Similar findings were reported by Stephensen et al. [34] and Mitra et al. [35], who found increased urinary losses of VA derivatives in severe illness, supporting the hypothesis that declining VA levels may reflect both pre-existing deficiency and acute depletion during critical illness.

Although our analysis supports routine screening of VA status in septic children, it does not yet justify the consideration of off-label VA supplementation as a low-cost adjunctive therapy in clinical practice, underscoring the need for a well-designed interventional randomized controlled trial to assess the clinical impact of timely VAS in pediatric sepsis. Such a trial should prioritize children with confirmed VAD and sepsis, standardize dosing (e.g., WHO guidelines for VAD), and assess adverse outcomes, particularly mortality rate, organ dysfunction scores, and inflammatory marker trajectories.

Remarkably, WHO-recommended VAS programs have been shown to reduce child mortality by up to 34% in deficient populations [36], with meta-analyses confirming a 24% reduction in all-cause mortality among supplemented children aged six months to five years [37].

### Strengths and limitations

This study addresses an underexplored yet clinically relevant topic: the prevalence and prognostic implications of vitamin A deficiency in pediatric sepsis. A key strength lies in its prospective design, inclusion of a well-defined control group, and the comprehensive evaluation of clinical outcomes in a homogenous cohort of critically ill children. The study also employed standardized diagnostic criteria for sepsis and utilized robust methods for vitamin A quantification. However, several limitations should be acknowledged. First, the single-centre design and relatively modest sample size may limit the generalizability of the findings and introduce potential selection bias. Second, the cross-sectional nature of the study and the reliance on a single serum vitamin A measurement at admission, without serial assessments to capture temporal fluctuations during the disease course, further complicate causal inferences between VAD and clinical outcomes. Consequently, it remains unclear whether the observed deficiency represents a pre-existing nutritional state or an acute phase response secondary to sepsis. Third, the analysis's unadjusted nutritional status, despite the well-established relationship between malnutrition and VAD, represents a potential confounder that could affect the observed associations. Forth, some inflammation and nutritional markers, which could potentially influence vitamin A levels, were available only for patients with sepsis and not for healthy controls. As we are concerned about the potential impact of the pathophysiology of acute illness itself, which may confound the interpretation of these markers. Additionally, baseline vitamin A status prior to illness onset was not available, precluding definitive conclusions about causality between VAD and sepsis progression. Furthermore, the study did not evaluate the therapeutic impact of vitamin A supplementation on patient outcomes, which remains an important area for future clinical trials. These limitations underscore the need for larger, multicentre studies with longitudinal follow-up and interventional components to fully elucidate the role of vitamin A in pediatric sepsis.

## Conclusion

This study highlights a high prevalence of vitamin A deficiency among critically ill pediatric patients with sepsis, with lower vitamin A levels significantly associated with greater disease severity and adverse clinical outcomes. While VAD was not independently predictive of mortality in this cohort, its correlation with multiple markers of illness intensity suggests a potential role as a prognostic biomarker in pediatric sepsis.

Given the immunomodulatory properties of vitamin A and its plausible pathophysiological involvement in sepsis progression, routine screening for VAD may be warranted in this population. Vitamin A supplementation represents a potentially low-cost adjunctive intervention; however, further randomized controlled trials are needed to clarify its therapeutic utility, define optimal dosing strategies, and determine its impact on morbidity and mortality in critically ill children.

## Supplementary Information

Supplementary Material 1.

## Data Availability

Data will be provided upon request from the corresponding author.

## Abbreviations

VA: Vitamin A
VAD: Vitamin A Deficiency
VAS: Vitamin A Supplementation
RA: Retinoic Acid
PICU: Pediatric Intensive Care Unit
PRISM: Pediatric Risk of Mortality
CRP: C-Reactive Protein
WBC: White Blood Cells
ALT: Alanine Aminotransferase
AST: Aspartate Aminotransferase
MV: Mechanical Ventilation
AUC: Area Under the Curve
ROC: Receiver Operating Characteristic
OR: Odds Ratio
CI: Confidence Interval
ELISA: Enzyme-Linked Immunosorbent Assay
