# Supplementary figures and images for: Prevalence and clinical impact of Vitamin A Deficiency (VAD) in critically ill children with sepsis
Source: BMC Pediatr. 2025 Oct 13;25:810. doi: 10.1186/s12887-025-06143-0 (PMC12516869; doi:10.1186/s12887-025-06143-0)

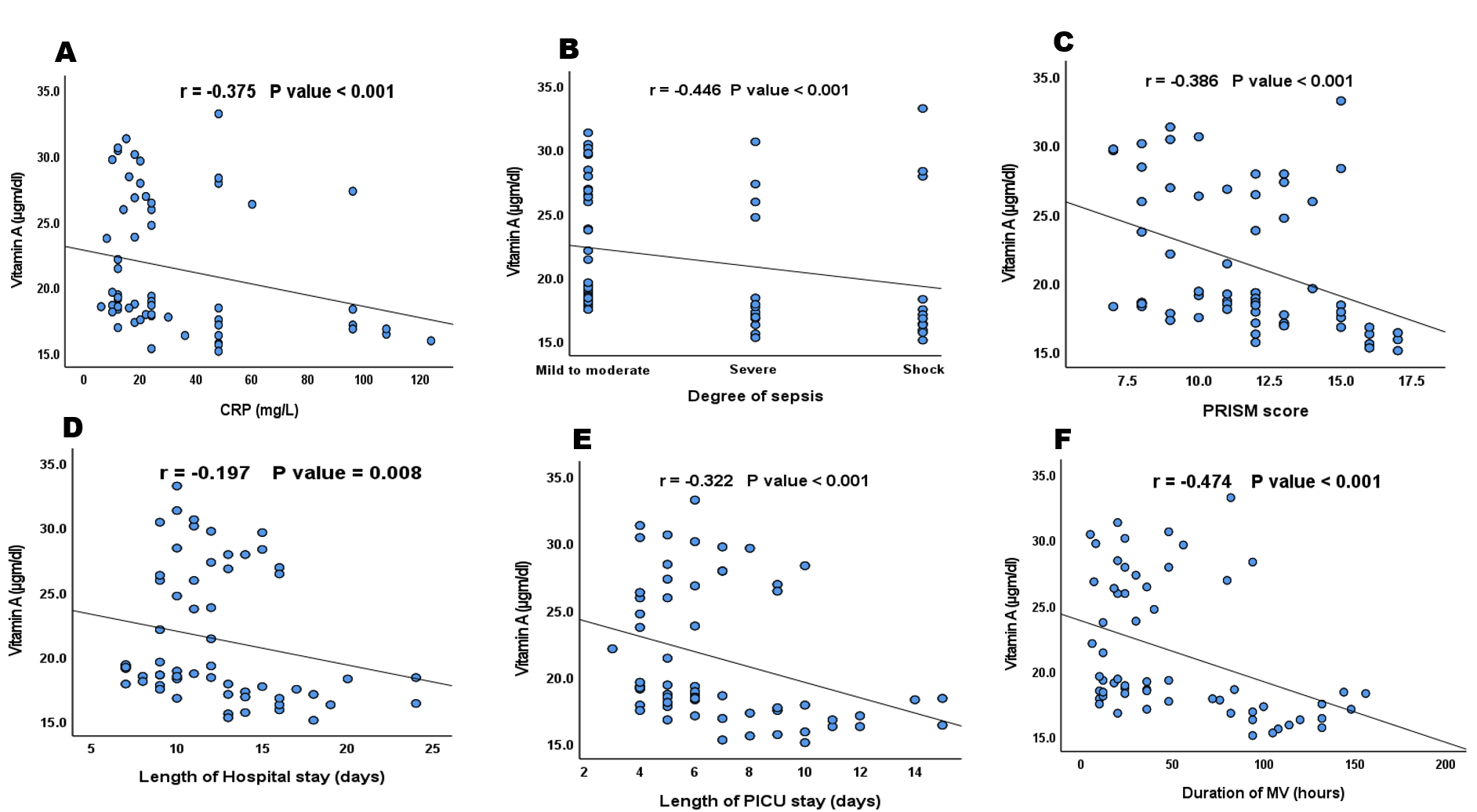

Supplement: Supplementary file 1 — Supplementary Material 1. [file 12887_2025_6143_MOESM1_ESM.png]
